# Regenerable ZnO/GaAs Bulk Acoustic Wave Biosensor for Detection of *Escherichia coli* in “Complex” Biological Medium

**DOI:** 10.3390/bios11050145

**Published:** 2021-05-07

**Authors:** Juliana Chawich, Walid M. Hassen, Céline Elie-Caille, Thérèse Leblois, Jan J. Dubowski

**Affiliations:** 1 Laboratory for Quantum Semiconductors and Photon-Based BioNanotechnology, Interdisciplinary Institute for Technological Innovation (3IT), Laboratoire Nanotechnologies Nanosystèmes (LN2), CNRS UMI-3463, Université de Sherbrooke, Sherbrooke, Québec, QC J1K 0A5, Canada; juliana.chawich@usherbrooke.ca (J.C.); mohamed.walid.hassen@usherbrooke.ca (W.M.H.); jan.j.dubowski@usherbrooke.ca (J.J.D.); 2 FEMTO-ST Institute, CNRS UMR-6174, Université de Bourgogne Franche-Comté, 25030 Besançon, France; celine.caille@femto-st.fr

**Keywords:** regenerable biosensor, bulk acoustic waves, piezoelectric ZnO thin film, GaAs membrane, self-assembled monolayers, bacteria detection, *Escherichia coli*

## Abstract

A regenerable bulk acoustic wave (BAW) biosensor is developed for the rapid, label-free and selective detection of *Escherichia coli* in liquid media. The geometry of the biosensor consists of a GaAs membrane coated with a thin film of piezoelectric ZnO on its top surface. A pair of electrodes deposited on the ZnO film allows the generation of BAWs by lateral field excitation. The back surface of the membrane is functionalized with alkanethiol self-assembled monolayers and antibodies against *E. coli*. The antibody immobilization was investigated as a function of the concentration of antibody suspensions, their pH and incubation time, designed to optimize the immunocapture of bacteria. The performance of the biosensor was evaluated by detection tests in different environments for bacterial suspensions ranging between 10^3^ and 10^8^ CFU/mL. A linear dependence between the frequency response and the logarithm of *E. coli* concentration was observed for suspensions ranging between 10^3^ and 10^7^ CFU/mL, with the limit of detection of the biosensor estimated at 10^3^ CFU/mL. The 5-fold regeneration and excellent selectivity towards *E. coli* detected at 10^4^ CFU/mL in a suspension tinted with *Bacillus subtilis* at 10^6^ CFU/mL illustrate the biosensor potential for the attractive operation in complex biological media.

## 1. Introduction

Bacterial infections present a critical public health problem worldwide, and the development of tools for rapid and reliable detection of pathogenic bacteria is one of the major challenges of modern medicine [1], environmental monitoring [2] and the food and water industries [3,4]. Rapid and sensitive detection of bacteria is a key factor of early identification and monitoring of diseases and may potentially prevent outbreaks and spread of grievous epidemics. *Escherichia coli (E. coli)* is one of the most abundant pathogenic bacteria regularly implicated in outbreaks of waterborne and foodborne infections [5,6]. Enterohemorrhagic *E. coli* (EHEC) can cause acute diseases such as gastroenteritis, hemorrhagic colitis and/or hemolytic uremic syndrome that can lead to kidney damage with a fatality rate of 3% to 5% [7]. In order to prevent these infections, it is necessary to detect and identify rapidly the presence of these bacteria at low concentrations. Conventional techniques for the identification and detection of bacteria include colony counting by culture, polymerase chain reaction (PCR) and enzyme-linked immunosorbent assays (ELISA). Although these techniques are highly selective and generally deliver decisive explicit results, they are considerably time-consuming and labor intensive [8].

The need for real-time detection has been a major driving force behind the emergence of biosensors as promising alternative platforms capable of rapid, sensitive and potentially cost-attractive detection of pathogenic bacteria [9]. Great effort has gone into developing new biosensors targeting *E. coli* [10,11,12,13,14,15] and limits of detection (LOD) as low as 1 CFU/mL have been reported for the detection of *E. coli* using different types of biosensors [16,17,18]. Despite the progress and the low LODs achieved in recent years, there is still no practical biosensor which could overall satisfy the market requirements, such as short analysis time, high sensitivity and aptitude for detection in complex media. Indeed, one major shortcoming of the available biosensors is the low sensitivity in complex detection environments (blood, serum, urine, contaminated water, etc.) [2]. For example, van Grinsven et al. [19] reported the detection of *E. coli* at 10^4^ CFU/mL by a biomimetic sensor using so-called surface-imprinted polymers (SIPs) [20], with a proof-of-application in a semi-complex matrix consisting of mixed bacterial solution containing both *E. coli* and *S. aureus* in a 1:99 ratio. More recently, Coudron et al. demonstrated the detection of *E. coli* with an estimated LOD of 2 × 10^7^ CFU/mL when present in a mixture of *HSA*, *Bacillus atrophaeus* (BG spores) and *MS2* bacteriophage [21]. Undeniably, working in complex environments can significantly affect the LOD for *E. coli*, as compared to pristine conditions, which raises a key challenge for the detection of this pathogen. As the simultaneous detection of *E. coli* in complex and real samples remains a major requirement, the biointerface selectivity and robustness become crucial to enhance the specific biodetection in complex media.

Acoustic wave biosensors have been of a particular interest due to their fast response and ease of design and fabrication, as well as their high sensitivity, their accuracy and stability [22]. Indeed, Micro-Electro-Mechanical Systems (MEMS) fabrication technology of acoustic waves-based sensors enables device miniaturization, power consumption reduction and integration with electronic circuits. Moreover, acoustic biosensors are attractive devices due to their relatively low cost of operation, while addressing the in situ detection of biomolecules in quasi-real-time.

Several materials are used for the fabrication of acoustic wave sensors. Among these, GaAs is a material that combines advanced MEMS technologies with possibilities of device integration and miniaturisation. The compatibility of this material with many chemical functionalization approaches and surface micro/nanofabrication processes makes it an ideal candidate for a biosensor application [23,24]. The surface of GaAs can be chemically functionalized with alkanethiols [25,26,27,28], silanes and phosphonates [29], and can be relatively easily regenerated [30,31], which gives this material very attractive functionalities for the fabrication of antibody-based architectures. Furthermore, it has been reported that an enhanced piezoelectric response of the GaAs-based acoustic sensor could be achieved upon deposition of a thin film of ZnO [32,33,34].

The present work reports the fabrication and testing of a label-free ZnO/GaAs-based bulk acoustic wave biosensor for detection and quantitation of *E. coli* in the presence of extraneous proteins. The antibody immobilization protocol was optimized to selectively capture *E. coli* on the sensing surface. A non-pathogenic form of *E. coli* was used as a bacterial model for detection tests to determine the dynamic range of the sensor and its LOD. The sensing responses were correlated with fluorescence images of the sensor surface for different concentrations of *E. coli,* and the regeneration of the biosensor was investigated over five cycles. The tests were performed in phosphate-buffered saline solution representing an ideal environment, and in the presence of highly concentrated *Bacillus subtilis* to simulate the detection in a “complex” biological environment.

## 2. Materials and Methods

### 2.1. Design and Operation of the Sensor

**Figure 1:**
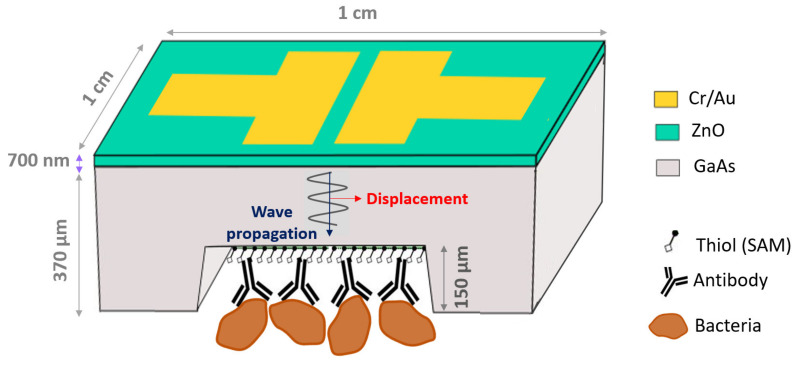
Architecture of ZnO/GaAs BAW biosensor (not to scale).

The geometry of the biosensor is shown in Figure 1. It consists of a 4 mm × 4 mm GaAs (100) membrane whose top surface is coated with a 700-nm thick film of piezoelectric ZnO. A pair of electrodes (Cr/Au, 20/200 nm) deposited on the ZnO film allows the generation of bulk acoustic waves in thickness shear mode, excited by lateral field and propagating in GaAs. The bottom surface of the membrane is biofunctionalized with alkanethiol self-assembled monolayers (SAM) and antibodies against *E. coli*. The chosen design has the advantage of separating the electrical interface from the biological fluid, which prevents the attenuation of the electric signal due to the potential contact with the liquid, and ensures frequency stability.

The Butterworth Van Dyke (BVD) model can be used to transcribe the behavior of the mechanical resonator to an electrical model [35]. The adhesion of biological elements on the surface shifts the resonance to lower frequencies and decreases their magnitude. Therefore, the quantitation of biomaterials adsorbed on the sensor surface is done by determining the shift in the resonant frequency, f_r_, associated with the mass variation on the surface. The added mass Δm of the biomaterial adsorbed on the surface can be calculated using the frequency shift ∆f_r_ at the fundamental frequency, as described by the Sauerbrey equation [36]:

(1) $$\Delta f_{r}=\frac{-{\;\Delta m\;f}_{r}}{A\;h\;\rho }$$

where A is the piezoelectrically active surface corresponding to the area between the electrodes (4 mm^2^), ρ is the density of GaAs (5.307 g/cm^3^) and h is the membrane thickness.

**Figure 2:**
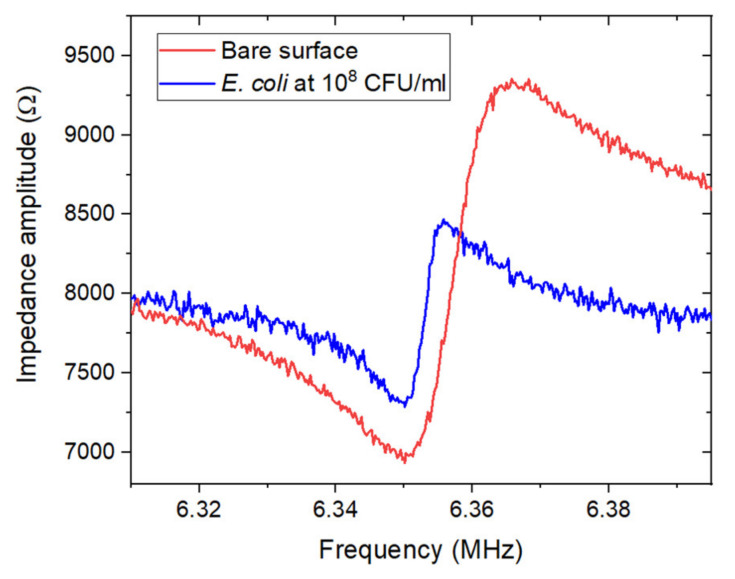
Example of the impedance amplitude measured near the resonant frequency (f_r_ = 6.35 MHz) for ZnO/GaAs membrane (220 μm) before and after exposure for 1 h to *E. coli* suspension at 10^8^ CFU/mL in PBS 1X.

The shifts in the resonant frequency were determined through electrical measurements, by connecting the Cr/Au electrodes of the sensor to an Agilent E5061B ENA series network analyzer (5 Hz–3 GHz) via a plug and play interface. The calibration of the analyzer was performed using the 85052D calibration kit, via its three standards corresponding to an ideal open circuit (zero admittance), an ideal short circuit (zero impedance) and a circuit with a load of 50 Ω. Figure 2 illustrates the resulting frequency shift after exposure of the biosensor to *E. coli* suspensions at 10^8^ CFU/mL for 1 h.

### 2.2. Materials

Undoped, 3-inch diameter and 625 ± 25 μm thick semi-insulating double-sides polished GaAs (100) ± 0.5° wafers (AXT, Inc., Fremont, CA, USA) were used to fabricate the biochips. Semiconductor-grade OptiClear (National Diagnostics, Atlanta, GA, USA), acetone (ACP Chemicals, Saint-Léonard, QC, Canada), anhydrous ethanol (Brampton, ON, Canada) and ammonium hydroxide (28%, Anachemia, Lachine, QC, Canada) were used as received. Deoxygenated ethanol (typically 40 mL) was prepared by flushing with a 3 SCFH high-purity nitrogen (99.9995%) stream (Praxair, Longueuil, QC, Canada) for 1 h. N-hydroxysuccinimide (NHS), 11-mercapto-1-undecanol (MUDO), 16-mercapto-1-hexadecanoic acid (MHDA), N-(3-dimethylaminopropyl)-N′-ethylcarbodiimide hydrochloride (EDC), phosphate-buffered saline (PBS) solution and ethanolamine hydrochloride were purchased from Sigma-Aldrich (Oakville, ON, Canada) and used without further purification. Unconjugated polyclonal IgG goat antibodies against *E. coli* and unconjugated polyclonal IgG rabbit antibodies against *Bacillus subtilis* were obtained from Virostat, Inc. (Portland, ME, USA). Deionized water at 18.2 MΩ.cm^-1^ was obtained with a Millipore purification custom system built by Culligan (Granby, QC, Canada). *Bacillus subtilis* ATCC 60514 (*B. subtilis*) and green fluorescent protein (GFP) *E. coli* K12 bacteria were provided by the Department of Microbiology of the Université de Sherbrooke Faculty of Medicine and cultivated in LB broth.

### 2.3. Microfabrication of ZnO/GaAs Sensor

Thin films of ZnO (~700 nm thick) were deposited on GaAs (100) substrates using a Plassys MP450S reactive radio frequency magnetron sputtering system employing a 6-inch metallic Zn target. Prior to deposition, the substrates were cleaned under an Ar and O_2_ plasma for 2 min and the target was pre-sputtered for 2 min to remove surface contaminants. During deposition, the substrate temperature and the gas pressure were 450 °C and 4 mTorr, respectively. The flows of Ar and O_2_, at 1 and 1.5 sccm, respectively, provided a deposition rate of approximately 2.77 nm/min. The full characterization and optimization of ZnO films on GaAs substrates was reported previously [37]. After the deposition of ZnO films, chromium/gold (Cr/Au, 20 nm/200 nm) electrodes were sputtered on the top using a lift-off process. Because of the difference in thickness between the desired membrane (50 μm) and the initial substrate (625 μm), the GaAs wafers were thinned down to 300 μm by chemical wet etching, which allowed an increase in the precision of the membrane machining. A layer of S1828 (MicroChem) resist was deposited beforehand on the front side of the substrate to protect the ZnO film and the electrodes during the etching. The thinning of the back side of the GaAs substrate was carried out in the etching solution of 7 H_3_PO_4_: 5 H_2_O_2_: 8 H_2_O at 0 °C, providing an etch rate of 0.85 μm.min^−1^ of the GaAs wafer [38]. Then, a standard S1823 resist layer (MicroChem) was deposited on the back side of the wafer by spincoating. After patterning, the substrate was immersed in the etching solution of 1 H_3_PO_4_:9 H_2_O_2_:1 H_2_O at 0 °C which provided an etch rate of 1 μm.min^−1^ [24]. After etching and monitoring the membrane thickness, the wafer was rinsed several times with deionized (DI) water, and the resist was removed by successive immersion in baths of acetone, DI water and ethanol.

### 2.4. Biofunctionalization of GaAs Membrane

**Figure 3:**
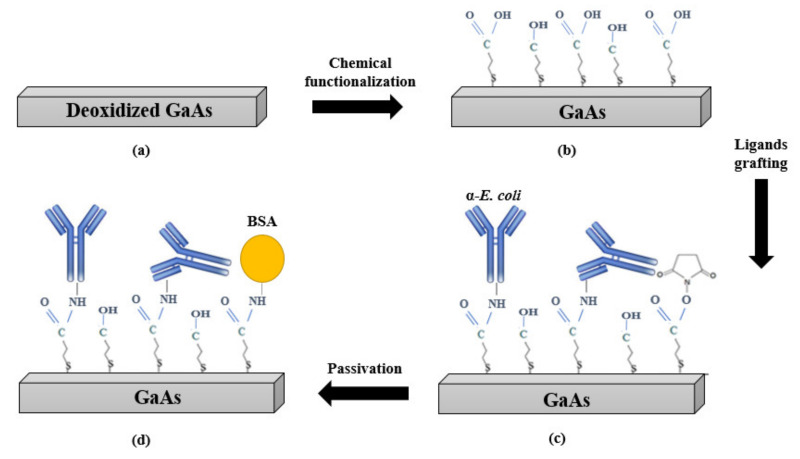
Schematic representation of the bio-functionalization stages: (**a**) bare and deoxidized GaAs surface, (**b**) MHDA/MUDO (1:9) chemical functionalization, (**c**) covalent immobilization of *E. coli* antibodies, (**d**) surface passivation with BSA and ethanolamine.

During biofunctionalization, the fabricated transducers were placed in a Teflon holder to expose only the membrane to the solutions while protecting the ZnO film and the Cr/Au electrodes. The biofunctionalization protocol is summarized as shown in Figure 3. First, the sensors were prepared for functionalization by cleaning in independent subsequent ultrasonic baths of OptiClear, acetone and ethanol for 5 min in each solvent. Then, the sensors were dried and etched to remove the native oxides by submerging them in 28% ammonium hydroxide for 2 min. Next, the sensors were thoroughly rinsed with deoxygenated ethanol and immediately incubated in 2 mM MHDA/MUDO (1:9 molar ratio) thiol solution for 20 h at room temperature. After incubation, the sensors were rinsed thoroughly with deoxygenated ethanol, followed by ultrasonic cleaning for 30 s in deoxygenated ethanol to remove the physisorbed thiols. Following the formation of the mixed SAM on the membrane surface, the COOH groups of MHDA were activated to provide covalent attachment with the antibody through an amide bond. The activation was conducted by incubation of the membranes in a mixture of NHS/EDC (1:4) at 1 mM for 30 min. After the activation, the excess of unreacted NHS and EDC molecules was removed by rinsing the samples 5 times with DI water. Then, the sensors were immediately incubated at room temperature in suspensions of *E. coli* antibodies diluted in different buffers (PBS 1X, pH 7.4 or acetate at pH 4.5) and at different concentrations (2.5; 12.5; 25; 100 and 200 μg/mL) for different durations (0.5; 1; 2; 4 and 20 h). Subsequently, the samples were thoroughly rinsed with PBS 1X containing TWEEN 20 (0.05% *v*/*v*). Then, the sensor surface was passivated by incubation in BSA solution (200 μg/mL, pH 5.5) for 30 min, and rinsed with PBS–Tween. Finally, the non-bound COOH groups of the processed samples were inactivated by incubation in ethanolamine (1 M, pH 8.5) for 30 min, followed by rinsing with PBS–Tween.

FTIR transmission spectra of antibody-functionalized samples were recorded to characterize the amide bands of the antibodies, immobilized under different conditions. The FTIR measurements were performed under vacuum with a Bruker Vertex 70v spectrometer equipped with a RockSolid interferometer and a wide-range Globar IR source covering 6000 to 10 cm^−1^. The spectra (512 scans) were collected with a liquid-nitrogen-cooled MCT (mercury cadmium telluride) IR detector, a spectral resolution of 4 cm^−1^ and an aperture of 1.5 mm. The spectrum of a freshly etched sample was used as a blank and subtracted from the samples spectra.

### 2.5. Immunocapture of E. coli on the Sensor Surface

The antibody-functionalized samples were placed in the Teflon holder in order to expose the membrane to *E. coli* suspensions ranging between 10^3^ and 10^8^ CFU/mL, prepared in PBS 1X following dilution of a freshly prepared culture in LB. The bacterial suspensions (typically 1 mL) were introduced manually into the holder using a pipette. After 1 h of static incubation, the bacterial solution was syphoned using a pipette then rinsed 3 times with DI water and dried under a flow of high-purity (99.999%) N_2_. Subsequently, electrical measurements were carried out (3 samples per each concentration of *E. coli*) to record the shifts in the resonant frequency. In parallel, fluorescence microscopy was used to characterize the density and distribution of bacteria on the surface, and to estimate the surface coverage with bacteria (3 samples per concentration of *E. coli*). The visualization of bacteria captured on the surface was performed using an Olympus IX71 fluorescence microscope equipped with a xenon lamp emitting at 470 and 490 nm. The microscope was connected to a DP71 digital camera and fluorescence images of the samples were taken by using Qcapture imaging software (QImaging Corporation, Surrey, BC, Canada). Six to eight images were collected per sample at different sites of the membranes with a 20× magnification. The fluorescence images were analyzed with ImageJ software [39] in order to estimate the corresponding bacterial surface coverage.

### 2.6. Sensor Specificity

To validate the specificity of the biosensor, two types of control experiments were prepared for the analysis, under nominally the same conditions. The first control concerned ZnO/GaAs membranes biofunctionalized with antibodies against *B. subtilis* and exposed for 1 h to suspensions of *E. coli* at concentrations ranging between 10^4^ and 10^8^ CFU/mL. The second control concerned ZnO/GaAs membranes biofunctionalized with *E. coli* antibodies and exposed for 1 h to *B. subtilis* suspensions diluted in PBS 1X at concentrations ranging between 10^4^ and 10^6^ CFU/mL.

### 2.7. Regeneration of the Sensor Surface

After each exposure to bacteria, the sensor surface was regenerated under acidic conditions of pH 2 using a commercial antigen–antibody dissociation kit from bioWORLD (Catalog No. 21310002-1 (650161)). The effect of the employed kit on the antibodies was investigated by measuring FTIR transmission spectra of the sensor biofunctionalized with *E. coli* antibodies, before and after exposure to the regeneration kit for 5 min. Moreover, the bacteria removal efficiency was investigated over 5 cycles of regeneration using fluorescence microscopy. In each cycle, the sensor was exposed to *E. coli* suspensions at 10^6^ CFU/mL for 1 h, then exposed to the regeneration kit for 5 min. After each exposure, fluorescence images were taken to monitor the surface coverage with *E. coli*.

## 3. Results and Discussion

### 3.1. Optimization of the Sensing Architecture on Bulk GaAs

#### 3.1.1. Optimization of the Concentration and Incubation Time for the Immobilization of *E. coli* Antibodies

The presence of antibodies covalently immobilized on the surface was studied by FTIR probing of the amide bands A, I and II located in the regions of 3296.4, 1644.4 and 1527.4 cm^−1^, respectively. According to Bandekar et al. [40], within the proteins, the amide band A is essentially due to the NH stretching vibrations, the amide I is rather associated with the C = O vibrations in stretching, whereas the amide II is related to the modes of torsion of the NH bonds as well as modes of stretching of the CN bonds. In this study, since the amide A peak was the most intense and least noisy peak among the amide bands, the antibody immobilization efficiency was evaluated by calculating the integrated absorbance intensity of the amide A (the area under the amide A peak) that was proportional to the total concentration of antibodies.

**Figure 4:**
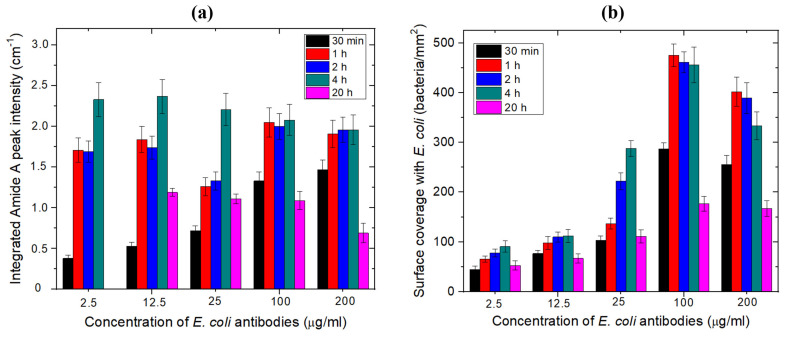
(**a**) Integrated FTIR amide A peak intensities for different incubation times and concentrations of *E. coli* antibodies prepared in PBS buffer (pH 7.4) on MHDA/MUDO (1:9) functionalized samples. (**b**) Surface coverage with *E. coli* bacteria immunocaptured on the biofunctionalized surface, determined by fluorescence microscopy for different concentrations and incubation times of *E. coli* antibodies (*n* = 3 samples per concentration and incubation duration).

Consequently, the values of the amide A integrating absorbance intensity were determined for each antibody concentration and incubation time at fixed pH 7.4, by using a Lorentz fitting, and reported by histograms shown in Figure 4a. At low concentrations of antibodies, the highest integrated absorbance intensities were obtained when the samples were incubated in *E. coli* antibodies for 4 h. However, when the concentration was increased, the integrated absorbance intensity values were comparable for incubation times between 1 and 4 h. The relatively low values of the integrated absorbance intensities observed for the 20-h incubation suggest that the antibody molecules deteriorated over long incubation durations. Thus, these results suggest that the 1-h incubation is optimal for the immobilization at a high concentration of antibodies.

The number of bacteria captured per mm^2^ for each antibody concentration and incubation time was obtained from the fluorescence images, as summarized in Figure 4b. It can be seen that, for low concentrations of antibodies, the highest density of bacteria was captured in the case of the 4-h incubation. However, for high concentrations (100–200 μg/mL), the number of captured bacteria per mm^2^ was comparable for the incubation time between 1 and 4 h, which is in a reasonable agreement with the FTIR data. Since the sample preparation time should be reduced as much as possible for a biosensor application, the antibody concentration and incubation time were set at 100 μg/mL and 1 h, respectively.

#### 3.1.2. Optimization of the Antibody-Grafting Buffer pH

**Figure 5:**
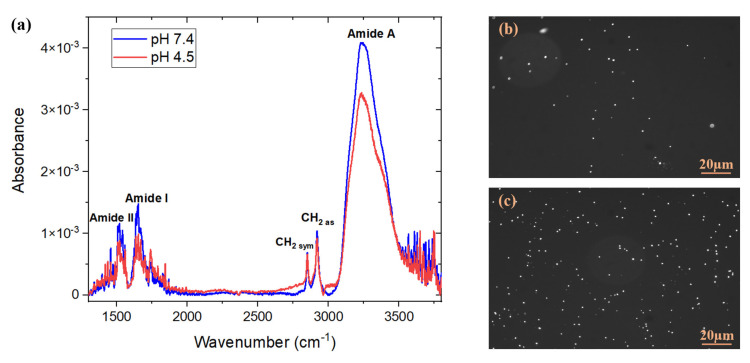
(**a**) FTIR absorbance spectra of *E. coli* antibodies immobilized on MHDA/MUDO (1:9) SAM-coated GaAs samples following 1-h exposure to 100 μg/mL antibody suspensions at two different pH conditions. Each spectrum represents an example of one of 3 tested samples; fluorescence images of GFP *E. coli* bacteria immunocaptured after 1-h exposure to *E. coli* suspensions at 10^6^ CFU/mL. The *E. coli* antibodies were immobilized following 1-h incubation in suspensions at 100 μg/mL in two different buffers: (**b**) acetate (pH 4.5) and (**c**) PBS (pH 7.4).

Examples of FTIR absorbance spectra measured after the immobilization of *E. coli* antibodies in PBS (pH 7.4) or acetate (pH 4.5), for the same concentration of 100 μg/mL and the incubation time of 1 h, are shown in Figure 5.

The integrated absorbance of the amide A feature over the range 3050–3550 cm^−1^ was estimated at 1.53 ± 0.07 cm^−1^ for the antibodies deposited in PBS at pH 7.4. This value was significantly greater than 1.22 ± 0.07 cm^−1^ determined for the same feature if antibodies were deposited in acetate buffer at pH 4.5. Thus, these results illustrate that PBS allows the immobilization of a higher number of antibodies.

Examples of fluorescence images of bacteria captured by the biosensor biofunctionalized with *E. coli* antibodies prepared in two different buffers and exposed for 1 h to *E. coli* suspensions at 10^6^ CFU/mL, are shown in Figure 5b,c. It was determined that the number of bacteria captured on the surface was 456 ± 24 bacteria/mm^2^ when the antibodies were prepared in PBS buffer, compared to 143 ± 16 bacteria/mm^2^ in the acetate buffer. These results are consistent with the FTIR findings and with the work of Pei et al. [41], who demonstrated that in a pH range from 2.5 to 8.5, the amount of antibodies that can be effectively attracted to the surface is the lowest at a pH below 5, since the charges on the surface start to decrease due to protonation of the carboxyl groups. Hence, PBS 1X at pH 7.4 was the buffer of choice for the immobilization of *E. coli* antibodies.

#### 3.1.3. Regeneration Efficiency of the Sensing Structure

The regenerability of the biointerface was tested after repeated exposure of the sample to bacterial suspensions. The approach consisted of detaching the bacteria but conserving the biointerface (i.e., the SAM and the antibodies) using the antigen–antibody dissociation kit. The FTIR transmission spectra of the amide bands, measured before and after exposure of samples biofunctionalized with *E. coli* antibodies to the regeneration kit for 5 min, indicated that the antibodies were not affected by the short-time exposure to the low pH environment of the regeneration. In fact, some enhancement of the integrated absorbance intensity of the amide A peak was observed after the exposure to the regeneration kit (Table 1), which suggests that the organisation of the antibodies on the SAM-coated surface was apparently improved. This result is in agreement with Djoumerska-Alexieva et al. [42] who demonstrated that a 5 min-exposure of IgG molecules to an acidic environment induced an enhanced binding efficiency of the investigated antibodies. It is relevant to notice that the CH_2_-vibration peak’s intensity and position remained unchanged during this procedure (1.0 × 10^−3^ ± 5.7 × 10^−5^ and 2919.5 ± 4.9 × 10^−3^ cm^−1^ for CH_2as_; 6.9 × 10^−4^ ± 7.1 × 10^−6^ and 2850.1 ± 9.5 × 10^−4^ cm^−1^ for CH_2sym_, respectively), which suggests that the thiols remained intact after the regeneration process. These results confirm that the tested regeneration kit and applied protocol preserve the functionality of the investigated antibodies.

**Figure 6:**
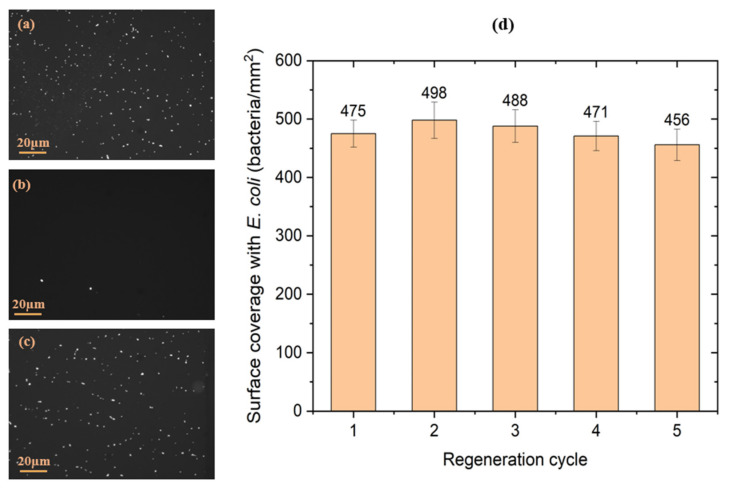
Examples of microscopic images of the antibody-functionalized GaAs biochips following the initial exposure to *E. coli* suspension (**a**)*,* after the 1st exposure to the regeneration kit (**b**) and after the 5th exposure to the regenerated kit followed by the exposure to *E. coli* (**c**). Evolution of the surface coverage with *E. coli* immunocaptured by the GaAs biochips (**d**) after each regeneration cycle (*n* = 3 samples).

**Figure 7:**
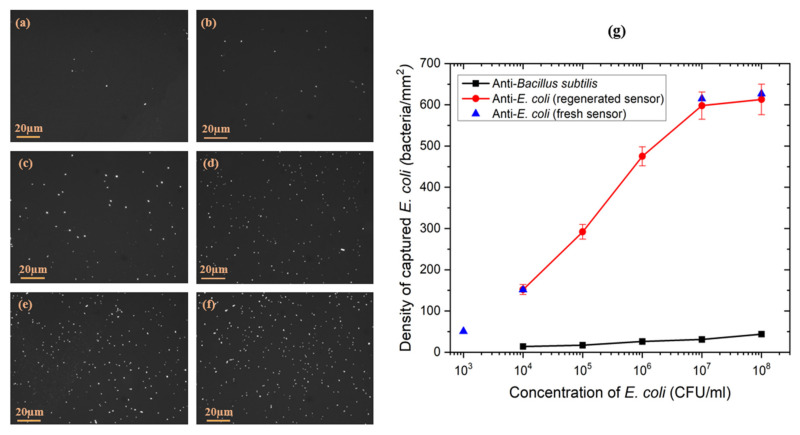
Examples of fluorescence images of GFP *E. coli* captured on the biofunctionalized GaAs surface of ZnO/GaAs biochips exposed to bacteria at (**a**) 10^3^ CFU/mL, (**b**) 10^4^ CFU/mL, (**c**) 10^5^ CFU/mL, (**d**) 10^6^ CFU/mL, (**e**) 10^7^ CFU/mL, (**f**) 10^8^ CFU/mL. Density of *E. coli* captured on the GaAs surface of the ZnO/GaAs biochip functionalized with antibodies against (**g**) *B. subtilis* (square symbols) or *E. coli* (circle and tringle symbols).

Examples of microscopic images of the antibody-functionalized GaAs biochips following the exposure to *E. coli*, the regeneration with a bacteria releasing kit and the repeated exposure to bacteria are shown in Figure 6. These results indicate the efficient bacterial removal and reproducible capture of bacteria after the regeneration. The initial capture of bacteria on the surface was at 475 ± 23 bacteria/mm^2^, and decreased to 10 ± 5 bacteria/mm^2^ following the removal step. After the second exposure to *E. coli*, the surface coverage increased to 498 ± 31, which is in agreement with the FTIR data that show increased intensity of antibody-related amide peaks following the exposure to acidic pH (Table 1). The density of captured bacteria remained in the limit of the error of the initial bacteria density, to finally reach 456 ± 27 bacteria/mm^2^ after the 5th exposure of the regenerated biochip to *E. coli* suspension. The evolution of the density of *E. coli* captured on the biochip surface is summarized in Figure 7d. These results clearly show the capacity of the investigated kit to regenerate the surface of the biosensor while preserving the efficiency of the biointerface for the capture of bacteria.

### 3.2. Determination of Antibodies and BSA Grafting Rates Using the ZnO/GaAs BAW Biosensor

The frequency shifts associated with the antibody immobilization and surface passivation with BSA (mass added to the surface) were determined theoretically and experimentally (Table 2). The theoretical shifts were determined by estimating the surface occupied by *E. coli* antibodies and BSA in saturation conditions. Since BSA is used to block the sites non-interacted with antibodies, the mass of BSA deposited on the surface should be inversely proportional to the immobilization efficiency of antibodies. The filling fraction of the 4 mm × 4 mm membrane was determined assuming that the minimal surface occupied by a single IgG *E. coli* antibody and BSA molecule was 34 [43] and 4.4 nm^2^ [44], respectively. The ratio of the experimental to theoretical shift for *E. coli* antibodies was between 56 and 79%, which suggests that up to 80% of the biochip surface was coated with *E. coli* antibodies. A slightly increased surface coverage with antibodies could still be achieved with a refreshable flow of an antibody solution and/or in the presence of an ultrasonic vibration [45]. As for BSA, the maximal surface coverage estimated was up to 50%, which is consistent with the role of BSA in blocking the sites that did not interact with an antibody.

### 3.3. Detection of E. coli Using the ZnO/GaAs BAW Biosensor

#### 3.3.1. Ideal Medium: Phosphate-Buffered Saline

The immunocapture of *E. coli* was quantitated by determining the frequency shifts measured after exposure of the biosensor to suspensions of *E. coli* at different concentrations. The number of captured *E. coli* per mm^2^ was determined by the ratio of the captured mass calculated using Equation (1), to the mass of one bacterium of *E. coli* at 9.5 × 10^−13^ g [46]. In these measurements, the biosensor was exposed to *E. coli* suspensions through cycles of regeneration/exposure, referred to as “regenerated sensor”, and with fresh surface referred to as “fresh sensor”.

To assess the surface coverage with bacteria captured by the biosensor, fluorescence images were taken immediately after exposure to each suspension of *E. coli* followed by rinsing three times with DI water and drying. The fluorescence images showed an efficient capture of bacteria reported with homogeneous distribution for each concentration of *E. coli*, as shown in Figure 7a–f.

Figure 7g shows the evolution of the surface density of bacteria for the different tested concentrations of *E. coli*. The change in the bacterial surface coverage between *E. coli* concentrations of 10^7^ and 10^8^ CFU/mL was almost negligible, indicating that the sensor reached the saturation stage.

**Figure 8:**
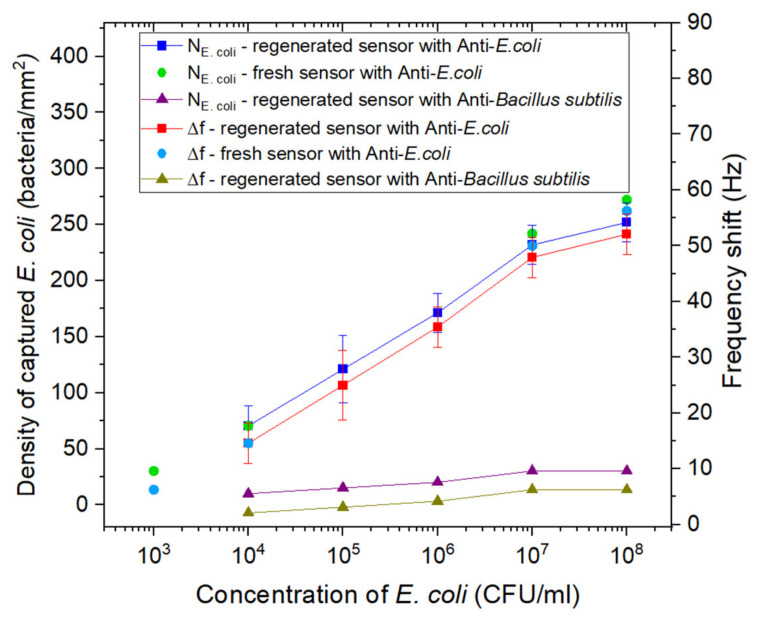
Surface density of captured *E. coli* (left *y*-axis) and frequency shifts (right *y*-axis) reported after exposure to increasing concentrations of *E. coli*: fresh sensor samples functionalized with *E. coli* antibodies (circles), regenerated sensor samples functionalized with *B. subtilis* antibodies (triangles), and regenerated sensor samples functionalized with *E. coli* antibodies (squares). The *E. coli* suspensions were prepared in PBS 1X.

The recorded frequency shifts and density of *E. coli* captured after exposure of fresh and regenerated sensors biofunctionalized with *E. coli* antibodies or *B. subtilis* antibodies to different concentrations of *E. coli* suspensions are shown in Figure 8. The measurement range of the biosensor was between 10^3^ and 10^8^ CFU/mL, with a linear region (log scale) between 10^3^ and 10^7^ CFU/mL. The LOD of the biosensor was determined at 10^3^ CFU/mL, and the curve representing the frequency shift Δf versus the logarithm of the concentration of *E. coli* can be described using the following relation: Δf (Hz) = 9.77 log [*E. coli*]–23.61.

For the fresh sensor samples, slightly higher frequency shifts and bacteria density were recorded, but the values remained in the limit of the error of the frequency shifts and bacteria density obtained for the regenerated sensor. This result is in reasonable agreement with the data presented in Section 3.1.3, indicating the efficiency of the used regeneration kit in regenerating the surface of the biosensor while preserving the biointerface.

The samples functionalized with *B. subtilis* antibodies showed very low frequency shifts upon exposure to *E. coli* suspensions, as compared with the shifts measured for the sensor samples biofunctionalized with *E. coli* antibodies, which validates the specificity of the biointerface towards *E. coli.*

By comparing the density of bacteria captured on the surface calculated from the frequency shifts using Equation (1) and from the fluorescence data, the resemblance in the dynamic of the corresponding curves is obvious, although the discrepancy between the densities is 1.5 to 2 times. The difference could be explained by the overestimation of the number of bacteria captured on the surface by fluorescence microscopy. According to Diaspro et al., fluorescent proteins generally exhibit multiple localized events from each single fluorophore, which could lead to an overestimation of the real molecule number [47]. These results are also consistent with Lisle et al. who demonstrated that fluorescent microscopy increasingly overestimated (15.0 to 99.3%) the true bacterial abundance [48].

#### 3.3.2. In the Presence of Bacillus Subtilis

**Figure 9:**
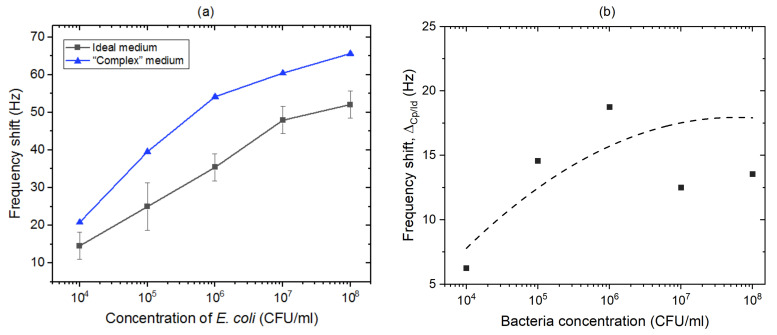
(**a**) Frequency shifts reported for the sensor exposed to increasing concentrations of *E. coli* in ideal medium (black squares), and *E. coli* in “complex” medium (blue triangles). (**b**) Difference between frequency shifts determined for *E. coli* in “complex” and ideal media (broken line is shown as a guide to the eye).

To simulate the detection of *E. coli* in the presence of another biological competitor (a “complex” medium), detection tests were carried out by exposing the biosensor functionalized with *E. coli* antibodies to a mixture of *E. coli* and *B. subtilis* in PBS 1X. The concentration of *E. coli* ranged between 10^4^ and 10^8^ CFU/mL, while the concentration of *B. subtilis* was constant at 10^6^ CFU/mL. Figure 9a compares the frequency shifts recorded for the biosensor exposed selectively to *B. subtilis* (red circles) and *E. coli* (black squares) suspensions in PBS (ideal medium) with the shifts determined for detection of *E. coli* in suspensions tinted with *B. subtilis* (“complex” medium).

The sensors functionalized with *E. coli* antibodies and exposed to *B. subtilis* suspensions showed lower frequency shifts compared to those induced by *E. coli* suspensions (~10 Hz at 10^6^ CFU/mL), which is clearly related to the expected selectivity of the biosensor. The non-specific signal could have been promoted by the static conditions of the experiments, and the perspective of using a refreshable flow of the bacterial solution using fluidic components should reduce it. The data representing frequency shifts versus the logarithm of the concentration of *E. coli* in “complex” medium follow the relation: Δf_r_ = 10.83 log [*E. coli*]–17.09. Furthermore, as shown Figure 9b, the difference in frequency shifts determined for “complex” and ideal environments, Δ_Cp/Id_, increased with the increasing concentration of *E. coli*. This difference (a differentiation contrast) is expected to saturate with the increasing concentration of bacteria, consistent with the limited biosensing efficiency of the developed method. The weakest differentiation contrast observed at 10^4^ CFU/mL was due to the onset of a significant competition between targeted bacteria and the presence of extraneous proteins. The performance of the ZnO/GaAs biosensor (LOD 10^4^ CFU/mL) in “complex” medium where *E. coli* is overwhelmed by a 100-fold excess of of *B. subtilis* underlines the potential that the biosensor could have in comparison with its equivalent [19,21]. We argue that the regenerable detection characteristics of the proposed ZnO/GaAs biosensor, and the demonstration of a strong response at 10^4^ CFU/mL combined with the future engineering focused on the elimination of non-targeted bacteria from complex media, present a major step towards the successful development of an innovative biosensing platform attractive for rapid detection of bacteria in clinical settings.

## 4. Conclusions

In our endeavour to develop an attractive biosensing platform for rapid detection of bacteria, we have designed and investigated the functioning of a ZnO/GaAs-based bulk acoustic wave biosensor. The capture and specificity of detection of *E. coli* was carried out with the antibody biofunctionalized biochips (100 μg/mL for 1 h in PBS 1X at pH 7.4). The tests carried out for bacterial suspensions ranging between 10^3^ and 10^8^ CFU/mL allowed us to determine the surface coverage with *E. coli* consistently with the measured frequency shifts. The LOD was estimated at 10^3^ CFU/mL for the bacterial suspensions in PBS (ideal environment), and the semi-logarithmic response of the biosensor was observed up to 10^7^ CFU/mL. The specificity of the biointerface to *E. coli* was validated through the control experiments involving *B. subtilis* bacteria at 10^6^ CFU/mL. The successful demonstration of a 5-times regenerated biochip and selective detection of *E. coli* in suspensions tinted with *B. subtilis* at 10^6^ CFU/mL was demonstrated with an LOD of 10^4^ CFU/mL. The attractive sensitivity of the proposed device, capable of a rapid and repetitive detection of bacteria, has the potential to bring the innovative and relatively inexpensive ZnO/GaAs-based bulk acoustic wave technology to support diagnostics offered in clinical settings.

## Figures and Tables

**Table 1:** Amide A peak values of *E. coli* antibodies measured by FTIR before and after exposure to the regeneration kit (*n* = 3 samples).

Stage	Before Regeneration	After Regeneration
Absorbance	4.4 × 10^−3^ ± 2.5 × 10^−4^	5.5 × 10^−3^ ± 3.8 × 10^−4^
Integrated absorbance intensity (in the range of 3050 to 3550 cm^−1^)	1.53 ± 0.07	2.09 ± 0.07

**Table 2:** Theoretical and experimental frequency shifts (∆f) determined after each phase of the bio-functionalization procedure of ZnO/GaAs biosensor (220 µm thick membrane) operating at the resonant frequency of 6.35 MHz (*n* = 3 samples).

Phase	Entity	$\left|\Delta f\;\right|\;(\mathrm{Hz})$	
		Theoretical	Experimental
Antibodies grafting	*E. coli* antibodies	39.84	27.1 ± 4.5
Passivation	BSA	136.46	59.8 ± 11.3

## Data Availability

Not applicable.
